# Explosive Bullets

**Published:** 1898-05-21

**Authors:** 


### XPLOSIVE BULLETS.

What Surgeon-Major-General Hamilton says about explosive bullets is important, as showing how very greatly their effects exceeded those produced by modern projectiles, even when their construction is modified, as in the dum-dum bullet, and we cannot fail at once to see how desirable it was that their use in warfare should be prohibited as it was by the Geneva Convention. As is well known, the old Snider, which was a large expanding bullet, was a most deadly missile. It had a hollow in front which was filled by a plug of wood or clay, while the base was also hollowed and plugged. On impact it became completely expanded and deformed, causing most terrible injuries to soft parts as well as to bones. The next step was to leave out the plug in front, forming a hollow space there, over which the lead was drawn, so that there was a closed cavity filled with air. On impact this air was strongly compressed, and tore the bullet open, much as a small charge of powder would have done. The "smashing" power of this bullet was so great that it was adopted for sporting purposes. "On soft-bodied animals, such as tigers and panthers, its effects were wonderful, the biggest tiger often dropping dead to a single shot when well placed." The true explosive bullet, however, against which the Geneva Convention legislated was a still more terrible projectile, and when fairly struck with it no soft-bodied animal could do further mischief. The bullet was cast in two parts. The base was hollow, and was nearly filled with ordinary powder, then a piece of thin cloth was laid over the top and the apex was fitted in, and the two were closed together in a machine. Through the front portion of the conical bullet so formed there was a hollow, leading from the apex to the cloth over the powder. Into this hollow detonating powder was poured, the hole was closed with wax, and the affair was complete. On impact the detonating powder went off, but the gunpowder was sufficiently slow in exploding to allow the bullet to penetrate before it broke up. This was a typical form of explosive bullet, and most deadly in its effects. "I shot a great deal of heavy game with it in India," says Surgeon-Major-General Hamilton, "and never lost an animal I knew I had struck. I have seen a stag 15 hands high hit behind the shoulder collapse on the spot, as if its legs had turned into jelly. On one occasion I saved my life by killing a charging bear in dense jungle, dropping her within a few feet of me. In this case I observed smoke issuing not only from the wound, but also from the mouth and nostrils, and on opening the chest found the lungs and heart torn to pieces." Let us be thankful for the Geneva Convention, which prohibited the use of such missiles against man.

